# A plea for program evaluation in a pandemic

**DOI:** 10.36834/cmej.70331

**Published:** 2020-12-07

**Authors:** Russell Dawe

**Affiliations:** 1 Memorial University of Newfoundland, Newfoundland and Labrador, Canada

These are unusual times. On March 11, 2020 the World Health Organization declared the COVID-19 outbreak a pandemic. In the months that followed, medical educators and learners have been forced to adapt. Clinical service, research, learner assessment, and navigating education-career transitions are all changing. So much is now based on virtual platforms and new questions arise. How should we teach clinical assessment and patient-centred care when our learners are physically separated from their patients? How can we effectively assess learners’ clinical skills for one discipline when they’ve been redeployed to a different service to help manage the pandemic?

Evidence-based care and education are foundational to our profession. Nevertheless, when one sails in uncharted territory, it is difficult to follow a map. Thus, we innovate. Medicine has always been a field of innovation, advancing for the well-being of our patients and the populations we serve. However, this is the first time we have confronted this magnitude of global pandemic with the current technology enabling our rapid-pace collaboration and communication for the common good. We see examples of this as journals like the Canadian Medical Education Journal offer online forums for planning scholarly work, reducing redundancy and fostering teamwork across the continent and around the world. We see examples of this as conferences like the Canadian Conference on Medical Education shift to fully online methods of delivery so that scholarly discoveries in medical education can continue to be disseminated and discussed from our own homes.

Many are already researching the clinical prevention and management of COVID-19. However, let us not forget the fruit of scholarship waiting to be harvested from our medical educators’ innovations. As some work toward developing a vaccine, others must work to ensure we continue to train and graduate competent physicians, launching the next cohort of graduates with confidence into practice for the population awaiting them. This scholarship includes rigorous research in medical education but is by no means limited to it. Scholarly work, more broadly, includes the exchange of innovative ideas, particularly where accompanied by a critical assessment of those innovations’ successes or failures. If you have adapted your teaching or assessment methods or tools in a way that strengthens your residents, others can benefit if you share it.

My plea, therefore, is for program evaluation. A simple pattern consisting of innovation, evaluation, and dissemination can build a wealth of evidence in the medical education literature which will benefit medical schools and residency programs around the world. Journals, scientific committees of conferences, grant agencies, and other academic institutions are preparing to mobilize relevant scholarly work in a timely manner. We need program evaluation and, with technology facilitating new levels of collaboration and communication, we now have the opportunity to evaluate and share our creative approaches in a timelier fashion.

For some of us, the concept of program evaluation may seem daunting at first. However, the Plan-Do-Study-Act cycle of basic quality improvement is accessible and easy to learn.^1^ For a more rigorous approach, there are many excellent resources to guide us. The AMEE Guide No. 67 reviews logic models and a variety of approaches to program evaluation, along with the theory behind them.^2^ Durning et al describe a user-friendly 3-phase approach specifically for medical educators to evaluate their programs before, during, and after implementation.^3^ There are articles with ‘quick tips’^4^ and websites and blogs ready to provide support to would-be program evaluators, especially during this pandemic.^5^ However, of all the available resources, one can hardly do better than those provided by the Centers for Disease Control and Prevention’s “Framework for Program Evaluation in Public Health,” which clearly walks the reader through the 6-step process of (1) engage stakeholders, (2) describe the program, (3) focus the evaluation design, (4) gather credible evidence, (5) justify conclusions, and (6) ensure use and share lessons learned.^6,7^

At a time when much field research has been put on hold by social distancing, there may be researchers, program evaluators, residents, and students seeking scholarly ways to contribute to the fight against COVID-19. Medical educators are implementing so many new strategies in their programs, let’s take a scholarly approach and evaluate these changes to learn from them. Lessons learned can make one program stronger. Lessons shared can make all our programs stronger.
